# Supplementary material for: Fading regulation of diurnal temperature ranges on drought-induced growth loss for drought-tolerant tree species
Source: Nat Commun. 2023 Oct 30;14:6916. doi: 10.1038/s41467-023-42654-z (PMC10616191; doi:10.1038/s41467-023-42654-z)
Supplement: Supplementary file 1 — Supplementary Information [file 41467_2023_42654_MOESM1_ESM.pdf]

Supplementary Information

Fading regulation of diurnal temperature ranges on drought-induced growth loss for drought-tolerant tree species

Xianliang Zhang[1,2], Tim Rademacher[3,4], Hongyan Liu[2*], Lu Wang[2], Rubén D. Manzanedo[5]

[1]College of Forestry, Hebei Agricultural University, 071001 Baoding, China;

[2] College of Urban and Environmental Sciences, Peking University, 100871 Beijing, China;

[3] Institut des Sciences de la Forêt Tempérée, Université du Québec en Outaouais, J0V 1V0 Ripon, Québec, Canada;

[4] Harvard Forest, Harvard University, 01366 Petersham, Massachusetts, USA;

[5] Plant Ecology, Institute of Integrative Biology, D-USYS, ETH-Zürich, 8006, Zürich, Switzerland

[*]**Corresponding author**: Hongyan Liu, Peking University. Email: lhy@urban.pku.edu.cn

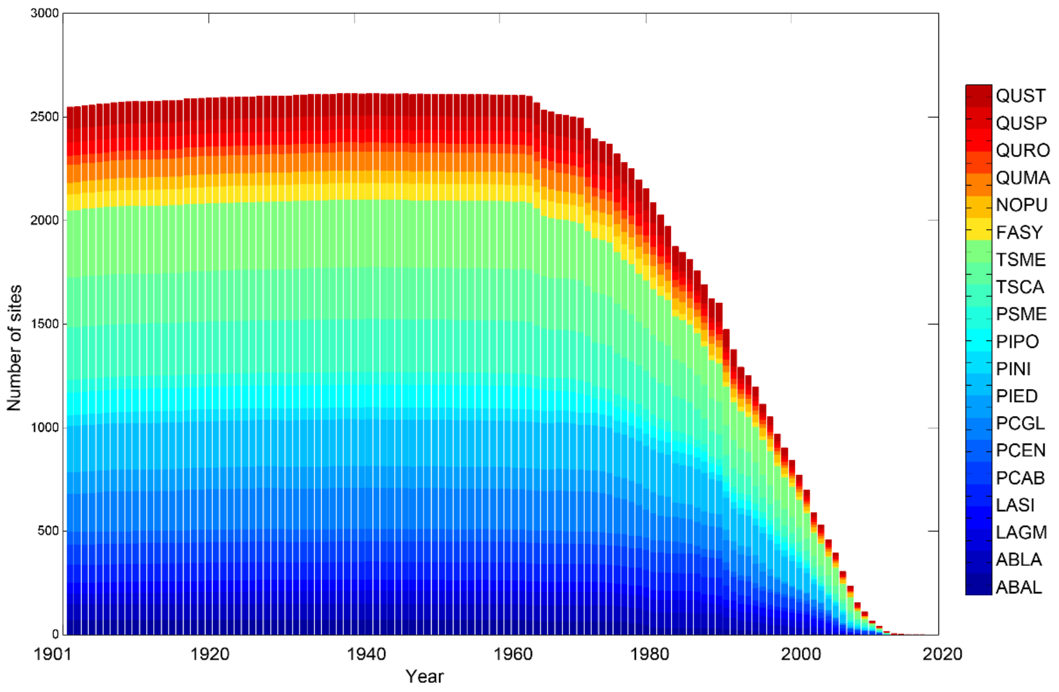

Fig. S1| Temporal changes in sample depth of different species. There are more than 2000 sites before the year 1980. Different colors represent different species.

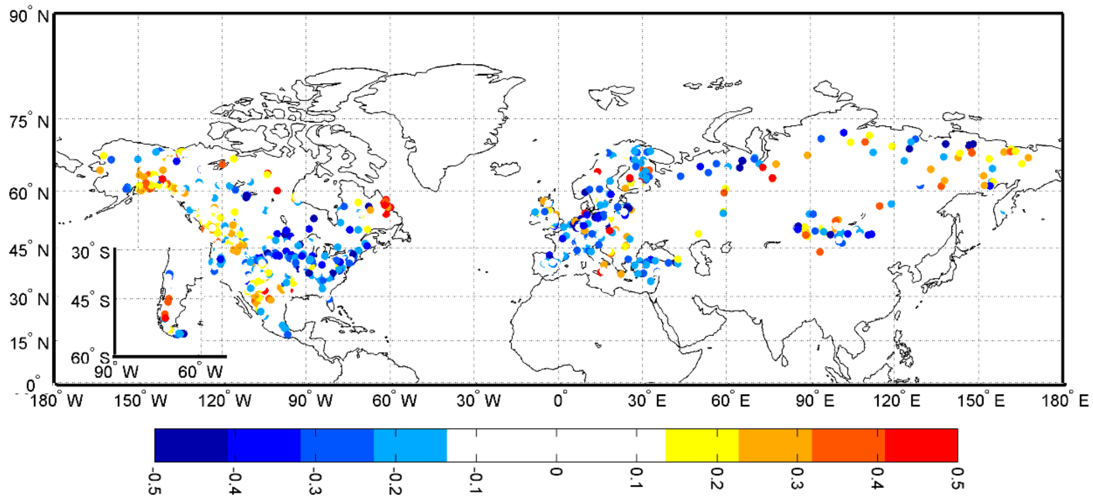

Fig.S2 | Spatial distribution of partial correlation between DTR and tree growth with the influence of temperature during dry years excluded.

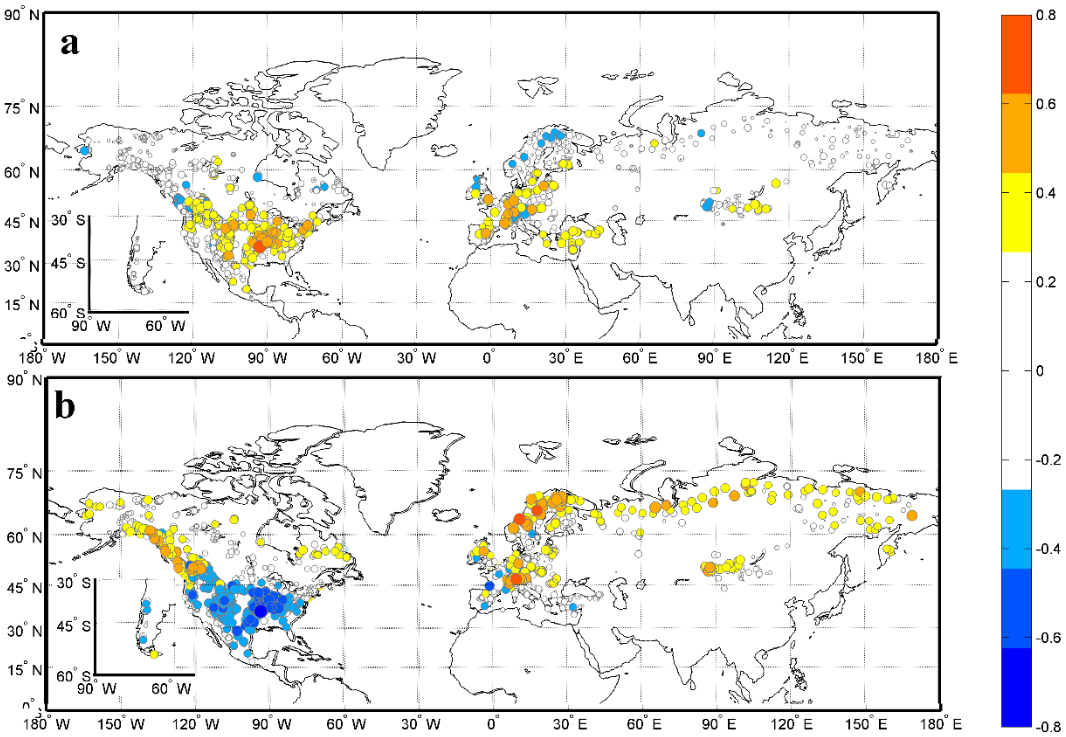

Fig. S3 | Correlation coefficients between tree growth and mean summer temperature (a), mean summer precipitation (b) for each site. The size of the cycle represents the size of the correlation coefficient.

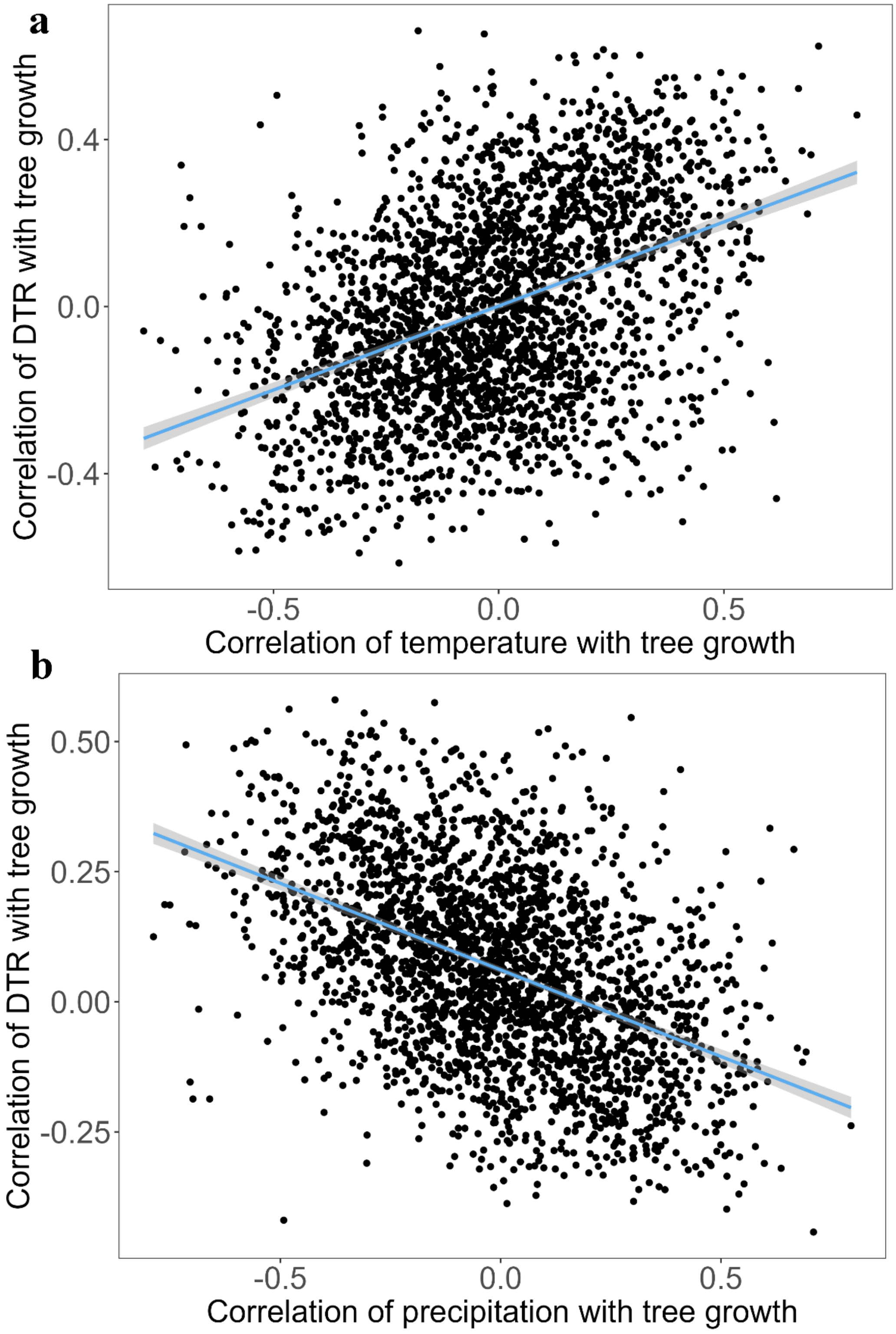

Fig. S4 | Relationships between DTR-growth correlation and growth sensitivity to temperature (a) and precipitation (b) during dry years.

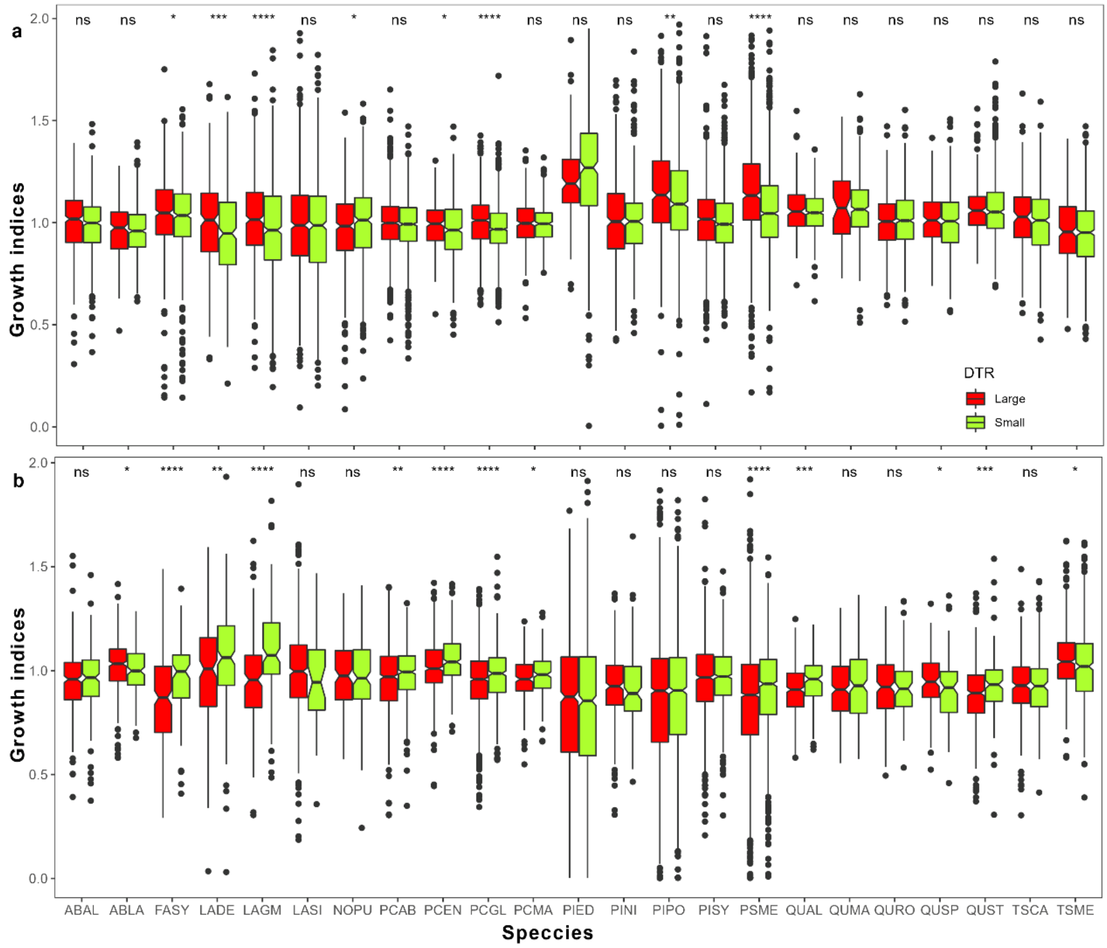

Fig.S5 | Comparison of tree growth indices between regions with high and low DTR small regions during wet (a) and dry years (b) for each species. *: p<0.05; **: p<0.01; ***: p<0.001; ****: p<0.0001; ns: non-significant by t-test.

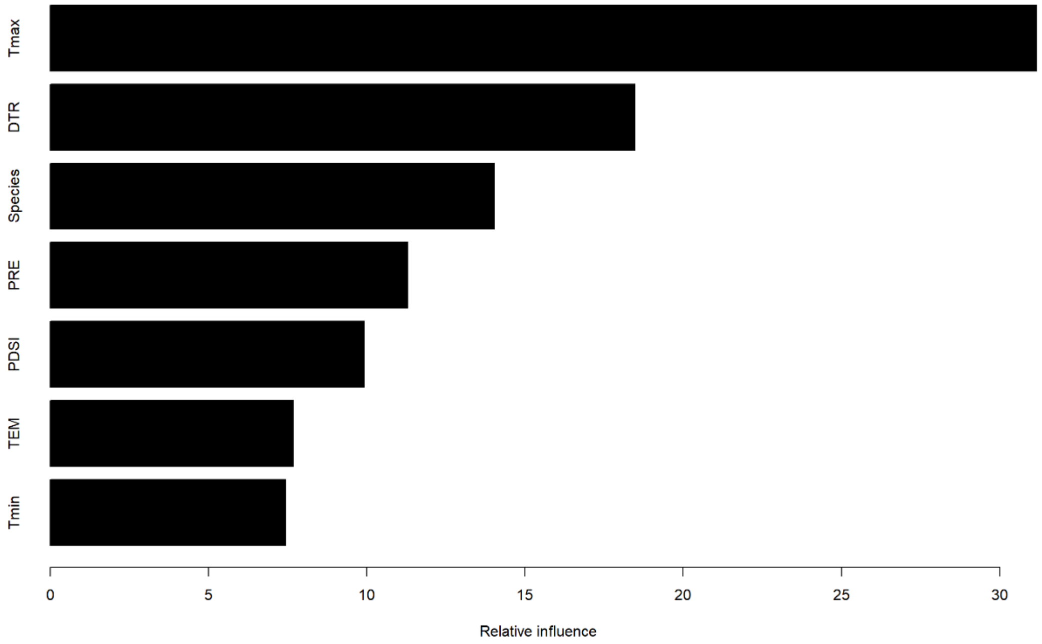

Fig. S6 | Relative contributions of different variables to drought-induced growth loss identified with boosted regression tree model. Tmax: maximum temperature, DTR: diurnal temperature ranges, PRE: precipitation, TEM: temperature, Tmin: minimum temperature.

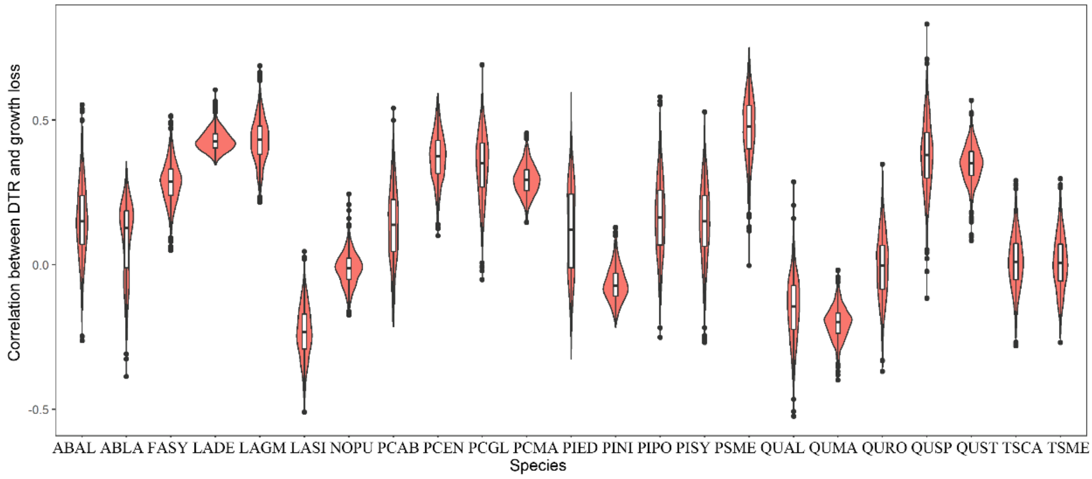

Fig. S7 | Correlation between DTR and growth loss during drought years for each species. Species ID can be found in Table S1.

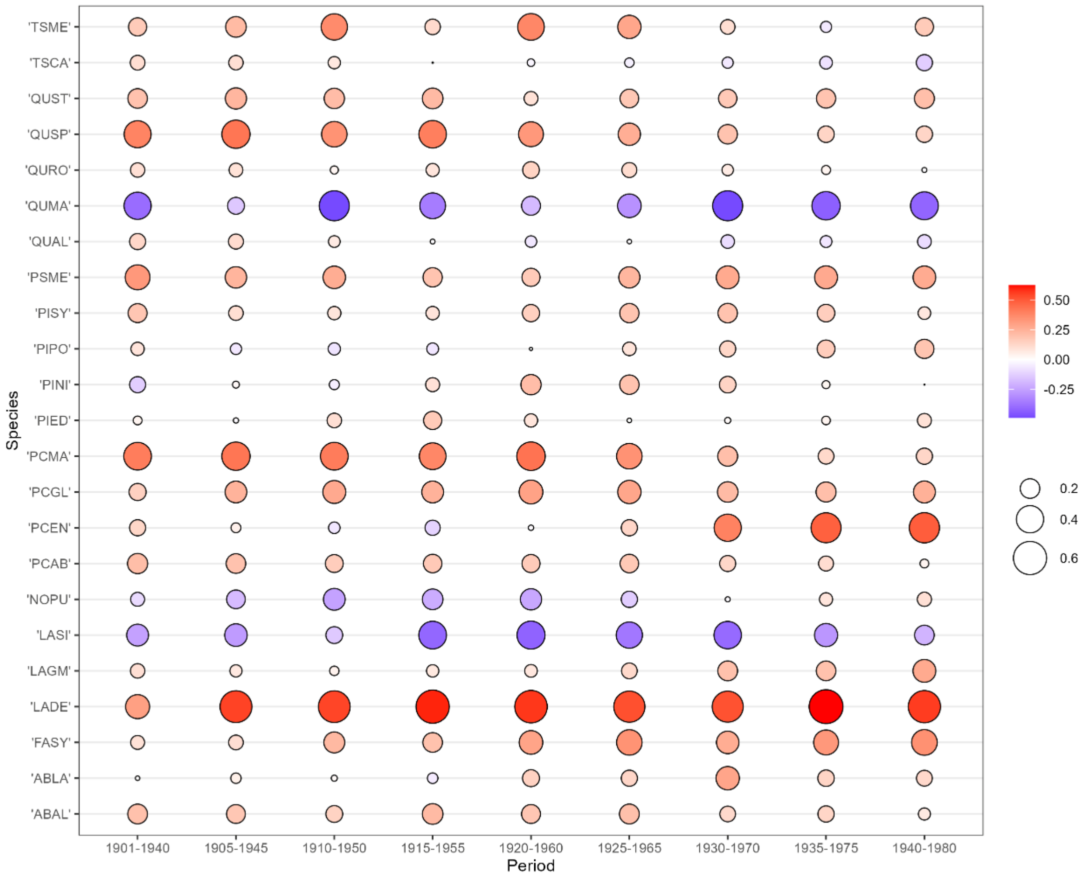

Fig. S8 | Temporal changes in the correlation between DTR and growth loss. Each solid circle shows the correlation coefficient between DTR and growth loss for a specific species. Species IDs are listed in Table S1.

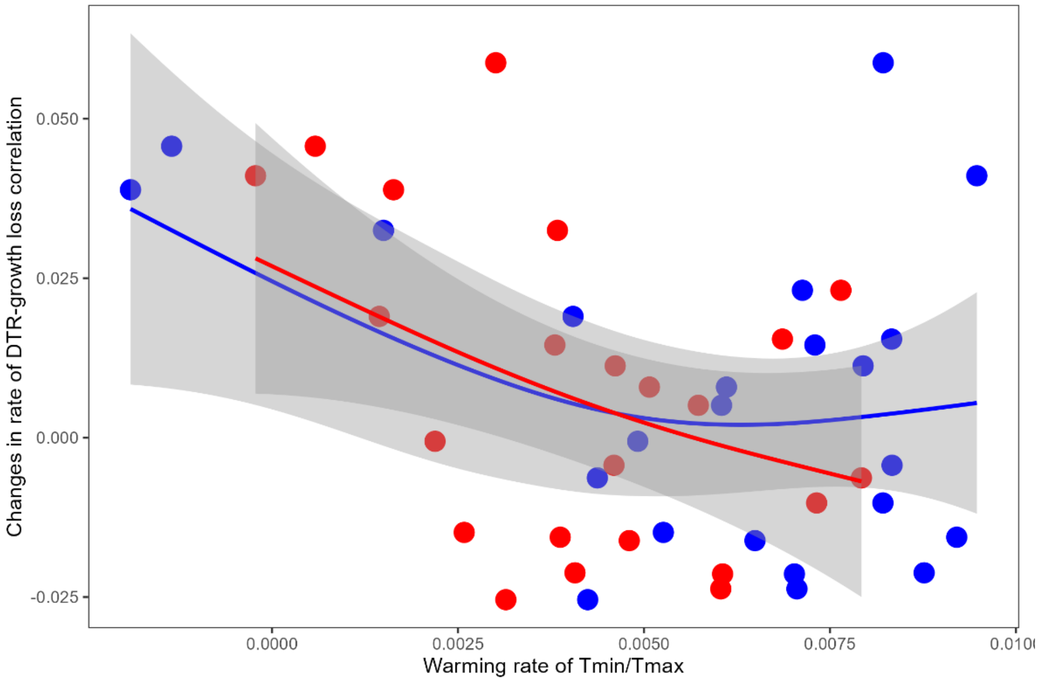

Fig. S9 | Relationships between changes in the rate of Tmin (blue)/Tmax (red) and changes in the DTR-growth loss correlation over time for the period 1901-1980.

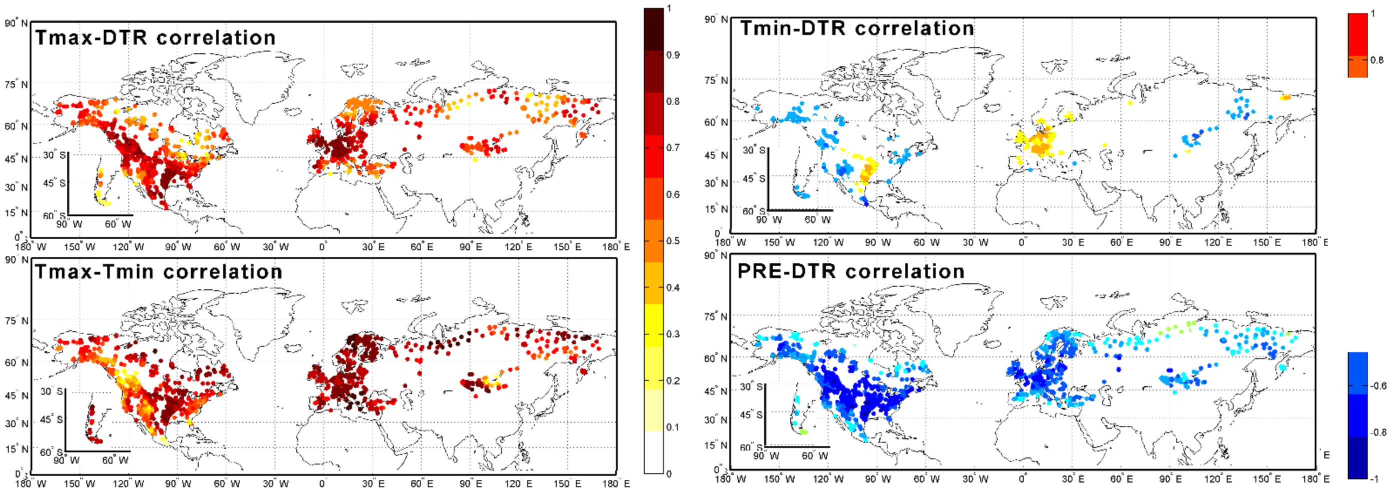

Fig.S10 | Correlations between DTR, Tmax, Tmin, and Precipitation (PRE) during 1901-1980 for all the sites.

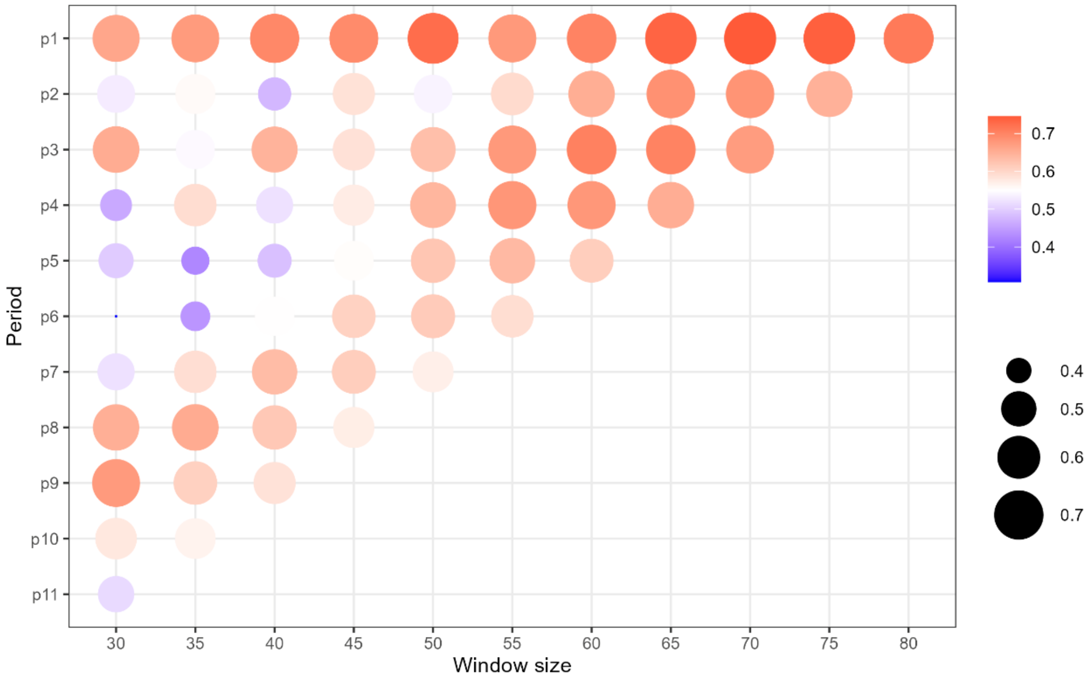

Fig. S11 | The sensitive test of window sizes from 30 to 80. P1 to p11 represent the first period with the initial year 1901 to the last period with the end year 1980.

Table S1| P_{50} for coniferous species and **P_{88}** for broadleaf species with references from which the hydraulic values were obtained.

ID	Species	P_{50}	P_{88}	Number of sites	Reference
ABAL	*Abies alba*	-3.65		44	Choat et al. 2012
ABLA	*Abies lasiocarpa*	-3.34		76	Choat et al. 2012
FASY	*Fagus sylvatica*		-3.80	40	Choat et al. 2012
LADE	*Larix decidua*	-3.66		44	Choat et al. 2012
LAGM	*Larix gmelinii*	-4.50		89	Choat et al. 2012
LASI	*Larix sibirica*	-3.00		100	Dulamsuren et al. 2018
NOPU	*Nothofagus pumilio*		unavailable	52	
PCAB	*Picea abies*	-3.98		133	Choat et al. 2012
PCEN	*Picea engelmannii*	-4.91		104	Choat et al. 2012
PCGL	*Picea glauca*	-4.30		217	Choat et al. 2012
PCMA	*Picea mariana*	-5.30		42	Choat et al. 2012
PIED	*Pinus edulis*	-4.88		109	Choat et al. 2012
PINI	*Pinus nigra*	-2.80		58	Choat et al. 2012
PIPO	*Pinus ponderosa*	-2.65		245	Choat et al. 2012
PISY	*Pinus sylvestris*	-3.61		215	Choat et al. 2012
PSME	*Pseudotsuga menziesii*	-4.50		317	Condo 2019
QUAL	*Quercus alba*		-2.60	71	Choat et al. 2012
QUMA	*Quercus Macrocarpa*		-2.00	41	Yin et al. 2014
QURO	*Quercus robur*		-3.46	64	Choat et al. 2012
QUSP	*Quercus spp.*		-5.00	40	Choat et al. 2012
QUST	*Quercus stellata*		-3.90	64	Maherali et al. 2006
TSCA	*Tsuga canadensis*	-3.07		60	Choat et al. 2012
TSME	*Tsuga mertensiana*	unavailable		102	

Table S2| Linear mixed model fitted for growth loss for different periods

Period	1901-1940	1905-1945	1910-1950	1915-1955	1920-1960	1925-1965	1930-1970	1935-1975	1940-1980
**Model**	GL~PDSI+DTR+DTR*PDSI+(1|species/age)								
**PDSI**	-0.06	0.09	0.10	0.10	0.17	0.18	0.23	0.25	0.30
**DTR**	0.21	0.14	0.17	0.13	0.18	0.21	0.18	0.17	0.21
**DTR*PDSI**	0.14	-0.06	-0.07	-0.09	-0.21	-0.22	-0.30	-0.38	-0.40
**Model**	GL~Tmax+Tmin+Tmax*Tmin*PDSI+(1|species/age)								
**Tmax**	0.44	0.33	0.45	0.45	0.47	0.48	0.40	0.36	0.39
**Tmin**	-0.07	0.05	0.12	0.16	0.01	-0.14	-0.16	-0.13	-0.18
**PDSI**	-0.51	-0.39	-0.37	-0.43	-0.30	-0.16	-0.18	-0.04	0.10
**Tmax*Tmin**	-0.08	-0.09	-0.25	-0.37	-0.20	-0.07	0.02	0.03	0.07
**Tmax*PDSI**	0.73	0.43	0.49	0.72	0.42	0.24	0.05	-0.24	-0.42
**Tmin*PDSI**	0.42	0.46	0.40	0.45	0.47	0.36	0.61	0.51	0.40
**Tmax*Tmin*PDSI**	-0.61	-0.52	-0.54	-0.80	-0.66	-0.49	-0.54	-0.32	-0.15
